# Movement Related Beta-Band Modulation with OPM-MEG: A Pilot Study

**DOI:** 10.1007/s10548-025-01150-x

**Published:** 2025-12-01

**Authors:** Tobias Sevelsted Stærmose, Jakob Udby Blicher, Sarang S. Dalal

**Affiliations:** 1 Center of Functionally Integrative Neuroscience (CFIN), Department Clinical Medicine, Aarhus University, Denmark, Building 1710, Universitetsbyen 3, 8000 Aarhus C, Denmark; 2 Department of Neurology, Aalborg University Hospital, Aalborg, Denmark

**Keywords:** Amyotrophic lateral sclerosis, ALS, MEG, OPM, Motor neuron disease, Cortical beta

## Abstract

Optically pumped magnetometers (OPMs) represent a significant advancement in magnetoencephalography (MEG), offering high sensitivity without cryogenic cooling and enabling flexible sensor placement. In this pilot study, we evaluated whether a small, zero-contact 16-channel OPM array can capture movement-related beta-band modulation (event-related desynchronization/synchronization; ERD/ERS) in healthy participants and explored feasibility in a single patient with amyotrophic lateral sclerosis (ALS). MEG responses to visually cued active and passive finger movements were recorded in a magnetically shielded room with the OPM array and separately with 306-channel superconducting quantum interference device (SQUID). Time–frequency analyses focused on beta-band activity across baseline, ERD, and ERS periods. In healthy participants, both OPM and SQUID successfully captured movement-related beta oscillations, with no significant differences between active and passive conditions or between measurement systems, based on non-parametric tests. In the ALS patient, movement-related responses were attenuated and more affected by artifacts in the OPM data compared with SQUID, limiting interpretability. Although movement artifacts were noted, the OPM system provided group-level results in healthy controls comparable to SQUID-based MEG, demonstrating its viability and potential for rapid, flexible deployment. These findings indicate that a compact zero-contact OPM array can reliably measure movement-related cortical beta activity and may offer a cost-effective alternative to cryogenic MEG systems. In ALS, however, the present results should be interpreted strictly as a feasibility demonstration, and larger patient cohorts will be required to establish reliability and clinical utility.

**Supplementary Information:**

The online version contains supplementary material available at 10.1007/s10548-025-01150-x.

## Introduction

Amyotrophic lateral sclerosis (ALS) is a progressive neuromuscular disease with a lifetime risk of approximately 1 in 350, which increases with age. In Denmark, the highest incidence is observed in individuals aged 70–79 years (Levison et al. 2024). ALS causes progressive degeneration of upper and lower motor neurons, leading to paralysis and ultimately death (Feldman et al. 2022).

Existing ALS treatment options are limited and largely ineffective, however the first disease modifying drug was recently approved in USA and Europe for ALS associated with the SOD1 mutation, highlighting the need for early diagnosis (Miller et al. 2022). As reliable biomarkers for ALS are lacking, diagnosis can take up to a year post-symptom onset, delaying therapeutic intervention (Goutman et al. 2022).

Neurophysiological tests, such as electromyography and transcranial magnetic stimulation (TMS), assist in diagnosis but lack specificity and reproducibility. The Gold Coast criteria currently represent the most widely accepted diagnostic standard (Goutman et al. 2022; Feldman et al. 2022; Shefner et al. 2020).

In the last 15–20 years, multiple attempts to identify reliable biomarkers for both diagnosis and prognosis have been described. These include fMRI studies (Konrad et al. 2002; Lulé et al. 2007; Agosta et al. 2013), TMS (Menon et al. 2015), EEG (Iyer et al. 2015) and MEG (Dash et al. 2024) (for a review, see McMackin et al. (2023)).

MEG detects the magnetic fields generated by dendritic post-synaptic currents. It has distinct advantages over EEG, particularly due to the minimal attenuation that magnetic fields experience when traveling through the skull, dura, and skin (Proudfoot et al. 2014).

Beta rhythms (~ 14–30 Hz) in the sensorimotor cortex change during active, passive, or imagined movements. Before movement begins, beta power decreases (event-related desynchronization, ERD), and after completion, beta power increases (beta rebound or resynchronization, ERS) (Stancák and Pfurtscheller 1995; Pfurtscheller et al. 1998; Pfurtscheller and da Silva 1999). The effects are very similar in passive movements (Parkkonen et al. 2015).

These beta-frequency changes are well-established, particularly in healthy individuals. Over the past decade, EEG and MEG studies have further characterized similar phenomena in both resting-state and movement conditions (Bizovičar et al. 2014; Notturno et al. 2023; Proudfoot et al. 2017; Stefan Dukic et al. 2024; Trubshaw et al. 2024).

Among key features in the movement-related beta band are ERD and ERS, which remain consistent within the same subject over time but exhibit large inter-subject variability (Espenhahn et al. 2017; Neuper et al. 2005).

### Optically Pumped Magnetometers (OPMs)

First demonstrated by Cohen in 1968 (Cohen 1968), magnetoencephalography (MEG) offers key advantages over EEG: magnetic fields are mostly unaffected by intervening tissues (Baillet 2017; Cooper et al. 1965; DeLucchi et al. 1962). Because the magnetic fields measured by MEG are perpendicular to the electrical current captured by EEG, the two methods can complement each other (Boto et al. 2019). Although EEG is more affordable and portable, it is heavily influenced by tissue conductivity, causing signal distortions. MEG, including OPM-based systems, measures magnetic fields less affected by the skull and scalp, allowing more precise source localization.

The advent of miniaturized optically pumped magnetometers (OPMs) (Happer and Tang 1973; Shah and Wakai 2013) (reviewed in Tierney et al.) represents a significant advancement in MEG technology (Tierney et al. 2019). Unlike superconducting quantum interference device (SQUID)-based systems, OPMs operate without the need for cryogenic cooling (Cohen 1972). Instead, they operate at room temperature, using the optical properties of alkali atoms to measure magnetic fields with femtotesla sensitivity. Their performance is comparable to SQUID-based systems—potentially (Hill et al. 2020) even better because OPMs allow for closer sensor placement to the scalp, theoretically enhancing signal sensitivity by up to seven-fold (Iivanainen et al. 2017).

OPMs offer several key advantages over SQUID-based systems: they are compact and lightweight down to just a few cubic centimeters, allowing for flexible sensor placement. This adaptability allows studies of regions like the cerebellum or spinal cord, which are harder to access with conventional helmet-based SQUID systems. The small size also enables OPMs to support customizable modular configurations tailored to specific research or clinical needs (Alem et al. 2023).

Their room-temperature operation removes the need for liquid helium and its associated infrastructure, substantially reducing operational costs.

### Feasibility of OPMs in ALS Studies

The integration of OPMs into ALS research presents unique opportunities. Their portability and scalability make them well-suited for longitudinal studies in diverse patient populations. A pilot study indicate that OPMs can record beta-band activity with accuracy comparable to conventional MEG (Hill et al. 2020). Their flexibility also facilitates data collection in populations (e.g., pediatric or severely impaired ALS patients) where traditional systems are impractical.

Advancements in OPM technology align with ongoing efforts to make MEG more accessible and clinically relevant. Offering a promising approach for the non-invasive diagnosis and monitoring of ALS.

### Study Goals

Both high-density EEG and conventional SQUID-based MEG require relatively complex setups, which can delay data acquisition. Our objective was to demonstrate that OPMs offer a simplified alternative while enabling reliable cortical measurements with a minimal number of sensors. Additionally, we aimed to explore the feasibility of a zero-contact approach to minimize artifacts.

Furthermore, we sought to assess the practicality of OPMs in ALS research, given that patients with ALS or other neuromuscular conditions fatigue quickly. Reducing the time from arrival to data collection is thus essential for these populations.

To evaluate this, we conducted a movement-related experiment comparing a conventional SQUID-based system with a 16-channel OPM system.

## Methods

### Participants and Ethics

This proof-of-concept study included four healthy right-handed male participants (mean age 43.5, range 31–55) and one male ALS patient in his mid-30 s (ALSFRSr: 42, no right upper-extremity weakness; exact age omitted for privacy reasons). Ethical approval was obtained from the Regional Scientific Ethics Committee (ID: 65059), and all participants provided written informed consent. All measurements were conducted at Aarhus University Hospital, Aarhus, Denmark. The current data is part of a larger study of which the SQUID data has been published (Preprint, 10.1101/2022.09.28.22280359).

### Data Acquisition

MEG data was recorded with a 306-channel SQUID system (Triux MEG system, MEGIN Oy) and a 16-sensor zero-field OPM system (Version 2, FieldLine Inc., Colorado, USA). All recordings took place in a dimly lit, three-layer, magnetically shielded room (Vacuumschmelze GmbH & Co. KG, Hanau, Germany). During the SQUID session, bipolar EMG recordings were obtained from the flexor digitorum profundus, extensor indicis, and right eye vertical electro-oculogram using disposable gel-filled electrodes (Neuroline 720; Ambu A/S, Denmark). Before SQUID data collection, each participant’s head shape was digitized using the Polhemus FASTRAK system (Vermont, USA), and four head position indicator coils were placed to track movement. A MEG-compatible accelerometer (ADXL335, Analog Devices, Inc., Massachusetts, USA) was mounted on the right index fingertip to record movement data. SQUID data were sampled at 1000 Hz using a 0.1–330 Hz band-pass filter. Head movement and noise artifacts were corrected with MaxFilter (v2.2.15; Elekta Oy, Helsinki, Finland).

OPM data were acquired using 16 single-axis optically pumped magnetometers (OPM, Version 2, FieldLine Inc., Colorado, USA and software; Fieldline Recorder v1.4.35) that measure the magnetic fields orthogonally to the scalp surface, with the magnetometers operating in closed-loop mode, sampled at 1000 Hz, and filtered with a 0.01–500 Hz band-pass filter. No supplementary measurements (EMG, head movement, accelerometry, or head digitization) were obtained during the OPM session.

**Fig. 1:**
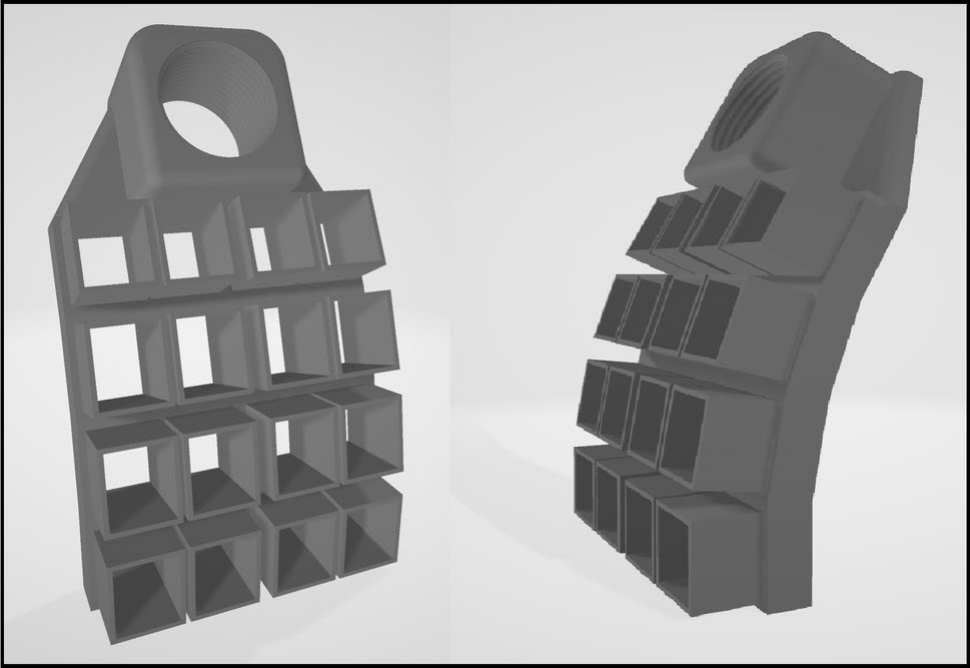
The 4 × 4 OPM array. Each square hole holds one OPM sensor, 16 total. The threaded hole (top round hole) fits a mounting arm to give flexibility in positioning

The SQUID system included a helium recycler with magnetic components, introducing a relatively strong gradient into the shielded room. For this reason, participants and the OPM sensors were placed on the side of the room opposite the SQUID system, where the background fields were the lowest. The gradient, however, implied that any sensor movement or vibration would result in strong motion artifacts. For this reason, a “zero-contact” holder was designed to hold the OPM sensors (see Fig. 1). The holder was fabricated using 3D-printed, carbon-fiber-infused polyamide (PA6/Nylon 6) (3DE Nylon CarbonForce, 3D Nordic Aps, Nørresundby, Denmark) specifically chosen to withstand any heat cycles the OPM would produce on the holder. Sensors was located via an individual slip-fit design to arrange the sensors in a 4 × 4 array (68 × 82.5 mm). The holder was designed to conform to a sphere approximating the average male skull size (Bushby et al. 1992). The holder was mounted on a ¾-inch Loc-Line adapter (Lockwood Products Inc., Oregon, USA), which was securely attached to a wooden floor stand with multiple mounting configurations (see Fig. 1).

This setup provided flexibility in positioning the array. During measurements, the array was placed as close as possible to the scalp above the contralateral sensorimotor cortex. The actual distance varied depending on hair thickness and subject movement. Participants were seated and rested their heads on an adjustable headrest to minimize fatigue.

### Experimental Design

Participants engaged in a movement task directed by a visual paradigm, performing both an active motor task involving the right index finger and a passive movement of the same finger facilitated by a pneumatic muscle (Fluidic Muscle DMSP, Festo, Germany). Pneumatic actuation was controlled by the Pneumatic Artificial Muscle Stimulator (Aalto NeuroImaging, Aalto University, Espoo, Finland) in a similar fashion to Piitulainen et al. (2015).

The movement of the finger consisted of a taping movement on a button. Participants were seated in an MEG-compatible chair with their arms resting on a table, with the active/passive button positioned slightly above the table surface to maintain a slight extension in the index finger throughout the experiment. To ensure consistent movement patterns, a rigid plastic guide secured the participant’s index finger, limiting motion to the metacarpophalangeal (MCP) joint by restricting distal joint movements. In both conditions, the vertical range of motion was consistently limited to about 2 cm. In the active movement the button press was damped by a block of cold expanded furniture foam, this gave a light resistance to compress the button. In the passive condition the button was contracted from below the table by the pneumatic actuator and moved the participant's index finger, replicating the motion pattern used during the active task.

Visual paradigm; Each trial comprised three phases designed to elicit a reliable ERD/ERS sequence. A 5–6 s fixation (gray cross) established baseline. This was followed by a 2 s preparatory cue: a yellow circle that filled radially toward the center. Participants were instructed to prepare the movement and to initiate the button tap precisely when the yellow fill reached the central target. Immediately thereafter, a 1 s action cue appeared: the stimulus turned green and filled outward to the outer edge, signaling the execution window and the return to baseline. For analysis, movement onset was time-locked to the moment the yellow fill reached the central target changing to the green cue. Each participant completed 100 active trials (five blocks × 20 cues, separated by four 30 s breaks) and 100 passive trials with the same schedule; during passive trials the same visual sequence was shown while the movement was imposed externally, and participants were instructed to remain relaxed and not assist. This timing minimized temporal jitter in movement onset and emphasized timing accuracy over force or speed.

Visual cues were presented via a PROPixx projector (VPixx Technologies, Saint-Bruno, Canada) on a display positioned roughly one meter ahead of the participant.

SQUID and OPM data were collected on separate days. Total recording time was approximately 45 min, with a preparation time of 30–45 min for SQUID and 5–10 min for OPM.

All participants, including the single ALS patient, completed both sessions.

### MEG Processing

*SQUID data preprocessing:* After applying MaxFilter to the SQUID data to correct for movement and external magnetic sources, we imported data into MNE-Python (Gramfort et al. 2013).

We selected 3 × 3 gradiometer pairs over the contralateral sensorimotor cortex, identified as the sensors with the highest beta-band activation.

Ocular and cardiac artifacts were removed using automated ICA (*.find_bads_eog,.find_bads_ecg*) (0–2 ICA components zeroed). The data were then epoched to the movement onset coded in the paradigm. After rejecting epochs exceeding 300 pT/m (peak-to-peak) (< 10% epochs removed), time–frequency representations (TFRs) were computed using discrete prolate spheroidal sequences (DPSS) from 3–120 Hz, windowed from − 4 to + 5 s around the movement.

*OPM data preprocessing*: Raw OPM data were imported into MNE-Python, and all channels were manually inspected for any obvious artifacts, no channels were removed at this step. The data were band-pass filtered between 8 and 45 Hz to remove low- and high-frequency artifacts. High-frequency projections were computed using mne.preprocessing.compute_proj_hfc.

We used ICA to further identify and remove artifacts by visual inspection of the ICA components (0–4 ICA components zeroed). Data was further inspected on a single channel basis, both as power plots, normalized power plots and time frequency plots, we only selected channels for exclusion if there showed complete detachment from the rest of the channels in all 3 domains (0–5 channels were rejected). After channel selection and artifact removal the data was epoched, in this step included rejecting epochs that exceeded 70 pT/m (peak-to-peak) (< 10% epochs removed) and extracted them from − 4 to + 5 s relative to the paradigm movement cue. Average (over trials and channels) TFRs were then calculated using DPSS from 10–40 Hz for each subject and task (active or passive).

For both SQUID and OPM; the TFR data (Frequencies * Time) were cropped to − 3.8 to + 4.5 s, then averaged across frequencies in the beta band (14–30 Hz) to yield mean beta power at each time–frequency point (Beta-power * Time). Finally, data were normalized (z-scored) to enable cross-method comparisons. The sight crop in the time window was to minimize spreading edge artifacts into the time points of interest.

### Statistical Analytics

Given the small dataset, we employed two main approaches: (1) assessing our method’s ability to differentiate beta power across three distinct time windows—baseline (rest), event-related desynchronization (ERD), and event-related synchronization (ERS); and (2) visually comparing OPM- and SQUID-derived data. Each participant’s average time–frequency representation (TFR) and beta-band power should exhibit broadly similar movement-related ERD and ERS features, although individual differences in timing, power, and frequency may occur. Notably, intra-subject features remain stable (Espenhahn et al. 2017; Illman et al. 2022).

A non-parametric Kruskal–Wallis (KW) test was conducted in R to assess the effects of Time Window (Baseline, ERD, ERS) (R Core Team 2022), Condition (Active, Passive), and Sensor Type (OPM, SQUID) on normalized mean beta power. This analysis included only the four healthy participants to avoid potential confounding effects from the ALS patient, as prior literature indicates beta-band alterations in ALS (Proudfoot et al. 2017; Trubshaw et al. 2024).

Mean normalized beta power values were calculated from three independent time bands. For active sessions: baseline (− 3.0 s to − 2.0 s), ERD (− 1.2 s to + 0.3 s), and ERS (+ 1.0 s to + 2.5 s). For passive sessions: baseline (− 3.0 s to − 2.0 s), ERD (− 0.5 s to + 1.0 s), and ERS (+ 2.5 s to + 3.8 s). These values were used for the KW test (see Fig. 3).

Three KW test models with mean beta power in the time windows as the dependent variable.


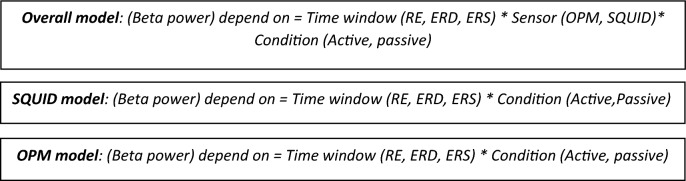


### Statistical Post-hoc Procedures

Following the primary Kruskal–Wallis (KW) tests on normalized beta-band power, we quantified effect size as η^2^_H using η^2^_H = (H − k + 1)/(N − k), where H is the KW statistic, k the number of groups, and N the total observations. To express precision, we computed 95% confidence intervals for η^2^_H by stratified bootstrap resampling (B = 2,000; sampling within groups to preserve group sizes). To contextualize sensitivity rather than to draw additional inferential conclusions, we converted η^2^_H to Cohen’s f ( f = √[η^2^/(1 − η^2^)]) and used a parametric ANOVA approximation (pwr.anova.test) to report the achieved power for the observed effect and the minimum detectable effect (MDE) at 80% power with the current sample size. All tests were two-sided with α = 0.05.

## Results

The Kruskal–Wallis overall model (Table 1) revealed a statistically significant effect of Time Window on mean beta power (χ^2^ = 41.80, p < 0.001), indicating that movement-related beta power varies significantly across the three time windows. In contrast, neither *Condition (χ*^*2*^ = 0.44, p = 0.5094) nor Sensor Type (χ^2^ = 0.01, p = 0.9343) showed a significant effect, suggesting no detectable difference between active and passive states or between the two sensor types (OPM vs. SQUID).

**Table 1:** Kruskal–Wallis test results

Test	Statistic	p
Time Window (Overall)	**41.80**	** < 0.001**
Condition (Overall)	**0.44**	**0.5094**
Sensor Type (Overall)	**0.01**	**0.9343**
Time Window (OPM Only)	**20.48**	** < 0.001**
Condition (OPM Only)	**0.40**	**0.5254**
Time Window (Squid Only)	**20.48**	** < 0.001**
Condition (Squid Only)	**0.05**	**0.8174**

*p* < 0.05 values marked in bold

*denotes statistical significance (α = 0.05)

### Sensor-Specific Analyses

OPM Model (Mean Beta Power ~ Time Window * Condition, within OPM data):

The effect of Time Window remained significant (χ^2^ = 20.48, p < 0.001), confirming that OPM detects time-dependent variations in mean power. However, Condition was non-significant (χ^2^ = 0.40, p = 0.5254), suggesting no difference was detectable between Active and Passive states.

**Fig. 2:**
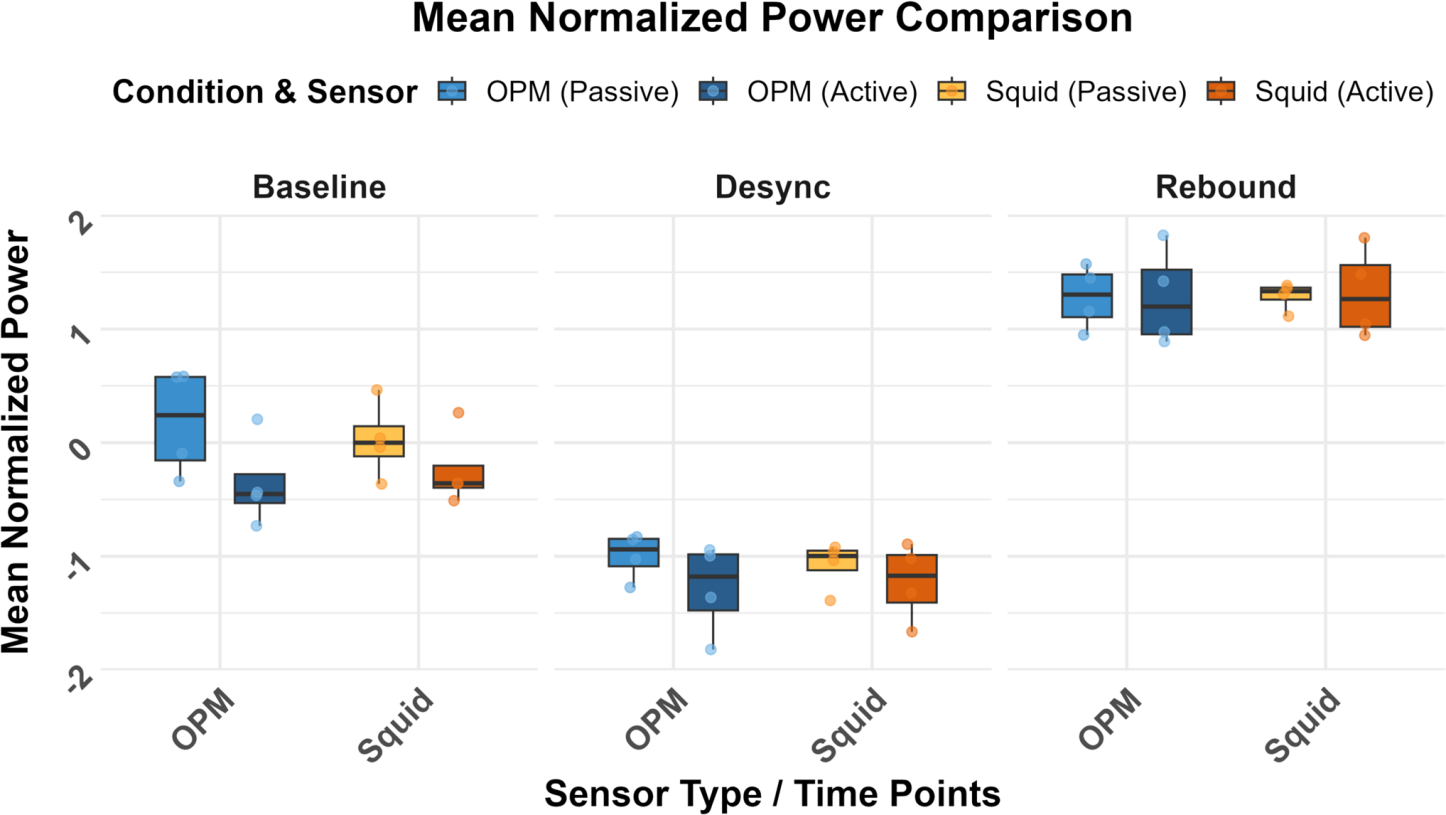
Boxplots showing distributions of the normalized mean beta power for the two different methods. Only data from healthy participants is included. Boxes show Interquartile range (IQR), Midline is Median, whiskers are 1.5*IQR, dots are individual values

SQUID Model (Mean Beta Power ~ Time Window * Condition, within SQUID data):

Similarly, the effect of Time Window remained significant (χ^2^ = 20.48, p < 0.001), confirming that SQUID also detects temporal differences in Mean Power. Condition remained non-significant (χ^2^ = 0.05, p = 0.8174), indicating no detectable difference between Active and Passive beta modulation. All results are shown in Table 1. The distribution of beta power values used in the Kruskal–Wallis test is visualized in Fig. 2 as a box plot.

In a post-hoc quantification of the Kruskal–Wallis results (Table 2), the time-window factor showed a very large effect on normalized beta power (η^2^ₕ≈0.88; 95% CI ≈0.88–0.89), and this held when analyzed within each sensor separately (OPM and SQUID η^2^ₕ≈0.88). Achieved power for this observed effect was ≈1, indicating the ERD/ERS modulation is robust and well detected in the present sample. By contrast, there was no evidence for main effects of condition (passive vs active) or sensor type (OPM vs SQUID): both had η^2^ₕ≈0 with upper 95% CI bounds of ~ 0.10 and ~ 0.09, respectively. A sensitivity analysis using a parametric ANOVA approximation showed that, with the current group sizes, the study had ≥ 80% power to detect moderate-to-large effects (Cohen’s f≈0.41; η^2^≈0.15) for condition or sensor. Together, these post-hoc estimates reinforce that the primary signal change is driven by the ERD/ERS time course rather than by acquisition condition or sensor technology.

**Table 2:** Kruskal–Wallis effect sizes and sensitivity

Test	H (df)	N	η^2^_H (95% CI)	Cohen f	Power	MDE f @80%	MDE η^2^ @80%	p
Time Window (Overall)	**41.80 (2)**	**48**	**0.884 (0.885–0.887)**	**2.77**	**1.00**	**0.46**	**0.18**	**0.0000**
Time Window (OPM)	**20.48 (2)**	**24**	**0.880 (0.883–0.892)**	**2.71**	**1.00**	**0.68**	**0.32**	**0.0000**
Time Window (SQUID)	**20.48 (2)**	**24**	**0.880 (0.883–0.892)**	**2.71**	**1.00**	**0.68**	**0.32**	**0.0000**
Condition (Overall)	**0.43 (1)**	**48**	**0.000 (0.000–0.103)**	**—**	**—**	**0.41**	**0.15**	**0.5094**
Sensor Type (Overall)	**0.01 (1)**	**48**	**0.000 (0.000–0.090)**	**—**	**—**	**0.41**	**0.15**	**0.9343**

For each factor we report H (df), sample size N, η^2^_H with 95% CI (stratified bootstrap), and Cohen’s f (derived from η^2^_H). Power is the post-hoc achieved power for the observed effect (ANOVA approximation). MDE = minimum detectable effect at 80% power (reported as f and η^2^). p from the Kruskal–Wallis test

*denotes statistical significance (α = 0.05)

**Fig. 3:**
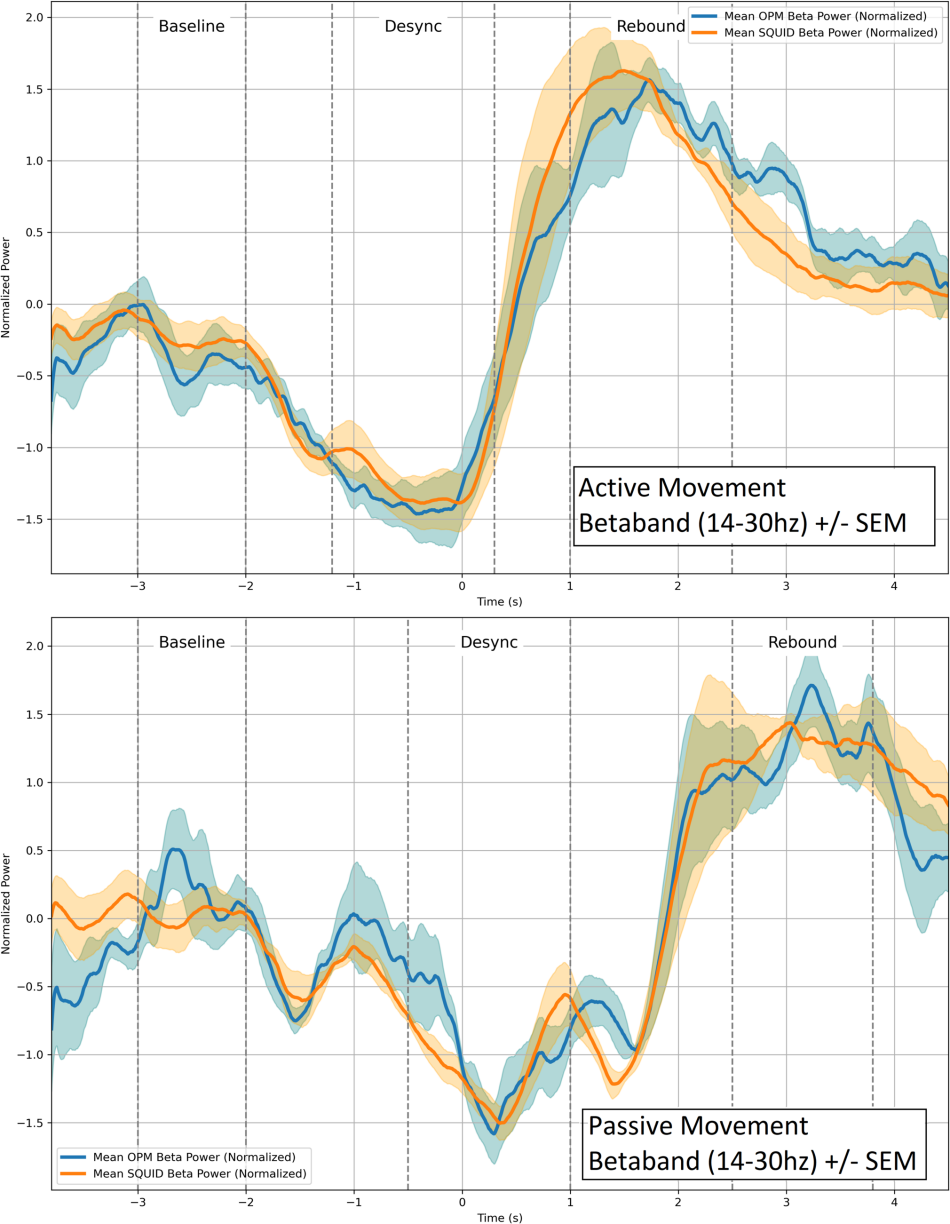
**(Top)**: Active movement data, showing mean z-scored beta power (14–30 Hz) (± SEM as shaded area) for all four healthy participants. The vertical lines indicate the time windows used in the Kruskal–Wallis test (rest, ERD, ERS). **(Bottom)**: Passive session, displaying mean z-scored beta power (14–30 Hz)) (± SEM as shaded area) for all four healthy participants. Time zero represents the movement cue in the paradigm, which was the same for both active and passive conditions

**Fig. 4:**
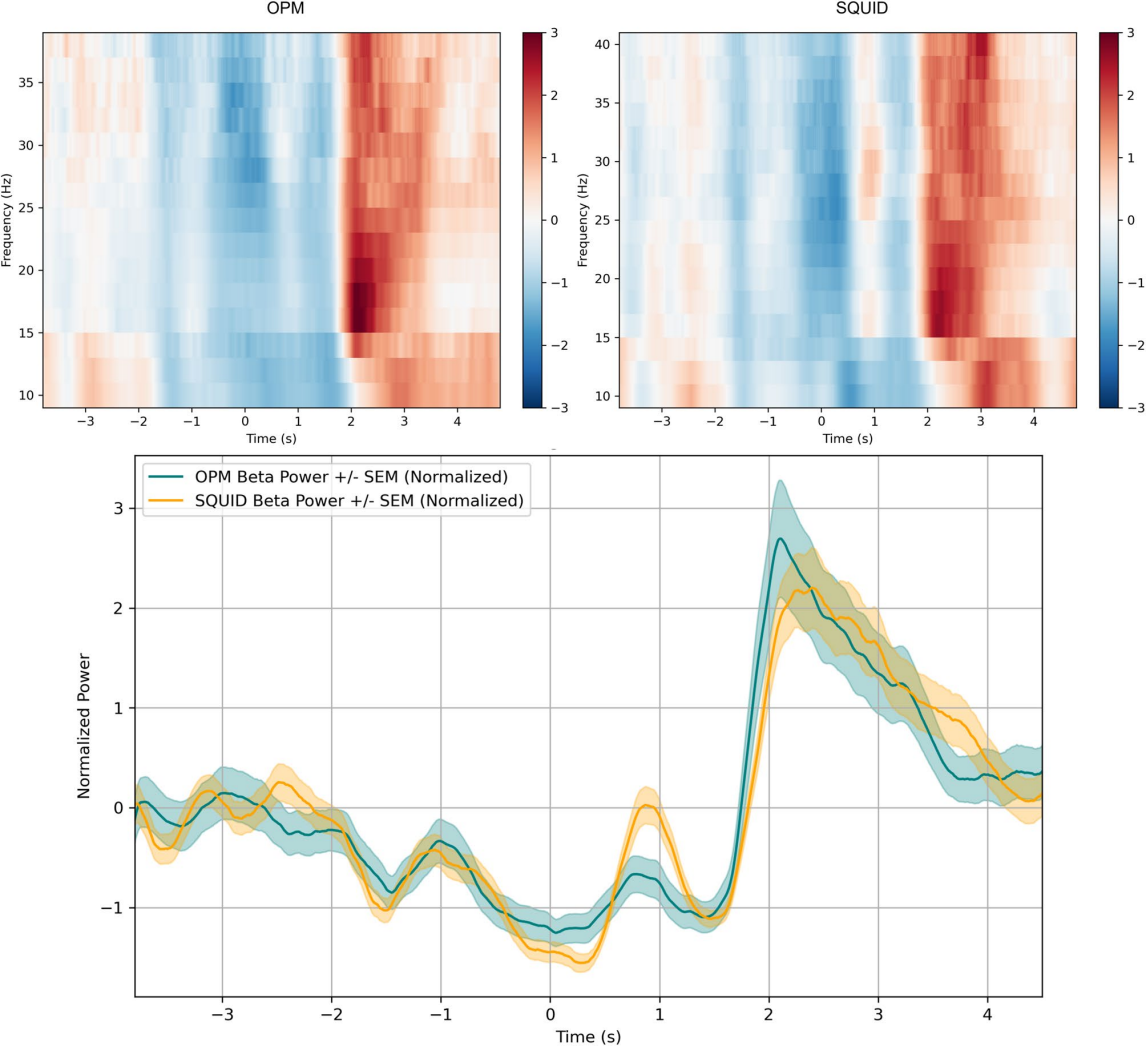
Responses for healthy participant 3 in passive condition. **Top Left:** Time–frequency representation (TFR) from the OPM session with passive movement, **Top Right:** TFR from the SQUID session with passive movement. Both TFRs are z-score normalized data. **Bottom:** Normalized beta power (14–30 Hz) comparison between the two methods (mean ± SEM(Shaded area))

To compare mean beta-band power across groups and assess the alignment between OPM and SQUID data, we computed averaged beta power (14–30 Hz) for both active and passive sessions (Figs. 3 and 4).

Figure 4 presents an example plot for a single participant, specifically Healthy Participant 3, in the passive condition. Both the TFR and beta power are displayed. Since the ALS patient was excluded from the statistical analysis, the ALS patient’s TFR and beta power output is presented separately in the supplementary Fig. 5.

A full catalogue of figures for all participant’s sessions and conditions are available in the supplementary material.

## Discussion

This exploratory study demonstrates that movement-related cortical beta signals can be reliably detected using a compact, 16-sensor OPM array that operates without physical contact with the participant. Despite the limited sample size, our results were comparable to those obtained with a conventional MEG system, indicating that the small OPM array effectively captures movement-related cortical beta activity. At the group level, the OPM system performed similarly to the SQUID-based system. Additionally, we demonstrate the feasibility of using this system for examining ALS patients.

Most prior human cortical OPM studies have used helmets, or flexible caps (Zhang et al. 2024), with the sensors placed in direct contact with the head, often with full-head coverage (Alem et al. 2023; Boto et al. 2017; Lin et al. 2019).

A primary motivation for developing the zero-contact array was to physically prevent movement-related artifacts in our measurements as much as possible. The shielded room contained a significant magnetic source, the helium recycler for the Triux SQUID system, increasing the OPM’s susceptibility to motion artifacts. Given the movement-related nature of our experiment, artifacts could still affect the results despite efforts to keep the OPM array off the participant’s head. We observed that even slight contact, such as hair brushing against the sensors, generated artifacts in this environment.

Our data revealed that many participants exhibited either movement-related artifacts or lower signal-to-noise ratios due to excessive sensor-to-scalp distance. Several participants displayed substantial movement artifacts that were difficult to fully eliminate during post-processing. Although the noise levels were higher in the OPM system compared to the SQUID system, we nevertheless observed robust and comparable activation patterns when averaging data from as few as four participants.

The ALS patient exhibited high levels of movement-related artifacts. However, we were still able to observe cortical signals that closely aligned with the much cleaner SQUID data.

A solution for the movement between sensor and scalp could be a more firmly locked head position but in all cases the distance to scalp is of the highest priority to achieve the best SNR and direct mounting would possibly be preferred if the environment allowed.

In our experiment, we also explored an additional advantage of OPM sensors: their simplicity and ability to enable rapid, single-modal, targeted cortical measurements. Our findings demonstrate that, without subject-specific devices or direct physical contact (e.g., sensors, gels, tape, helmets), it is possible to collect cortical data from the sensorimotor cortex at a quality comparable to that of an established SQUID system.

With further optimization, this approach could be particularly beneficial in scenarios where compliance, posture constraints, or fatigue limit data collection using conventional methods.

The minimal preparation time and high flexibility of this system could significantly benefit patients with limited mobility, as they tend to fatigue faster than healthy participants do. Fatigue is indeed a well-documented aspect of ALS (Jellinger 2024; Silva et al. 2024). In our study, preparation time required 30–45 min for the SQUID measurements, but under 10 for the OPM measurements.

However, the OPM data quality for the ALS patient in our study was poorer than for most of our healthy participants, most likely due to head and sensor movement in that particular session. Although beta power may be reduced in ALS (Bizovičar et al. 2014; Proudfoot et al. 2017; Trubshaw et al. 2024; Dukic et al. 2024) these previous studies report beta levels close to healthy participants (as an example the 2017 Proudfoot study show beta in ALS with a 10–20% difference range as the HC comparison group).

In future studies, OPM-MEG systems could be especially valuable for ALS patients approaching or experiencing a locked-in state. Notably, evidence of preserved motor cortical activity, even in late-stage ALS, has been documented in previous research (Aliakbaryhosseinabadi et al. 2021; Knudsen 2024).

Another key advantage of a compact system like the one presented is its lower acquisition and operational costs. With the appropriate setup and careful optimization, it is possible to obtain high-quality data using only a small number of OPM sensors, making the technique feasible for a larger number of research institutions and clinics.

### Limitations

The small sample size (four healthy participants and one ALS patient, all male) limits the generalizability of our findings. Our study did not seek to identify previously unexplored cortical features but instead focused on well-characterized event-related desynchronization (ERD) and event-related synchronization (ERS) in the beta band to validate the utility of OPMs to measure a common feature of movement-related brain activity relevant in ALS.

The limited sample size also necessitates caution in statistical analyses, as single-subject outliers may introduce bias and misinterpretation. In our Kruskal–Wallis test, we specifically excluded the ALS patient to minimize any ALS-related influence on the results as prior studies show alterations to beta in ALS (Trubshaw et al. 2024; Dukic et al. 2024; Proudfoot et al. 2017). The similar Kruskal–Wallis test statistics for the OPM and SQUID separate models suggest that both sensors provide a similar ranking structure of mean beta power across the three time windows. Since the Kruskal–Wallis test is rank-based, it does not compare absolute values but rather the relative ordering of data points. Given that both sensors capture the same underlying neural dynamics, their rank distributions follow a similar pattern, leading to identical test statistics. This finding supports the notion that OPMs and SQUIDs measure functionally equivalent signals in this context.

To enhance data quality in future work controlling and minimizing the array-to-scalp distance is crucial and may require redesigning the array.

A magnetically clean environment is undoubtedly optimal for OPM measurements. In the current study, the primary limitation was the presence of residual magnetic fields and gradients in the shielded room. A cleaner magnetic environment, possibly together with a magnetic compensation system, would allow the array to rest directly on the participant’s head both increasing signal strength due to closer proximity to the brain as well as better tolerating movements. Given the relatively small size of our array, sensor placement remains an important consideration. Here, we relied on well-reproduced literature and prior experience with sensorimotor movement–related beta activity to place sensors where signals were expected to be strongest.

## Conclusion

This proof-of-concept study shows that a compact zero-contact 16-sensor OPM array can capture canonical movement-related beta modulation (ERD/ERS) from sensorimotor cortex under our paradigm. Within the available healthy-participant data, OPM and SQUID produced comparable normalized beta-power patterns; we did not detect moderate-to-large differences between sensor types, although small differences cannot be excluded given the sample size and design. The single ALS case demonstrates feasibility of acquiring OPM-MEG in a patient with motor impairment, but it does not establish reliability or group-level effects in ALS; those claims await the larger cohort studies. Practically, the short setup and comfortable, flexible and contact-free mounting suggest that OPM-MEG may reduce participant burden relative to conventional MEG, making it a promising option for clinical research where posture, fatigue, or limited tolerance hinder data collection. Further work will validate these findings in larger patient and control samples and with improved arrays and artifact-reduction strategies.

## Supplementary Information

Below is the link to the electronic supplementary material.

Supplementary file1 (DOCX 7219 KB)

## Data Availability

Data are available upon request due to ethical and privacy considerations. Public sharing is restricted by ethical approval requirements. The code is available upon request by contacting the corresponding author.
