# Supplementary material for: Movement Related Beta-Band Modulation with OPM-MEG: A Pilot Study
Source: Brain Topogr. 2025 Dec 1;39(1):3. doi: 10.1007/s10548-025-01150-x (PMC12669360; doi:10.1007/s10548-025-01150-x)
Supplement: Supplementary file 1 — Supplementary file1 (DOCX 7219 KB) [file 10548_2025_1150_MOESM1_ESM.docx]

# *Supplementary Figures*

## *Figure 5: ALS Patient (Active Condition)*


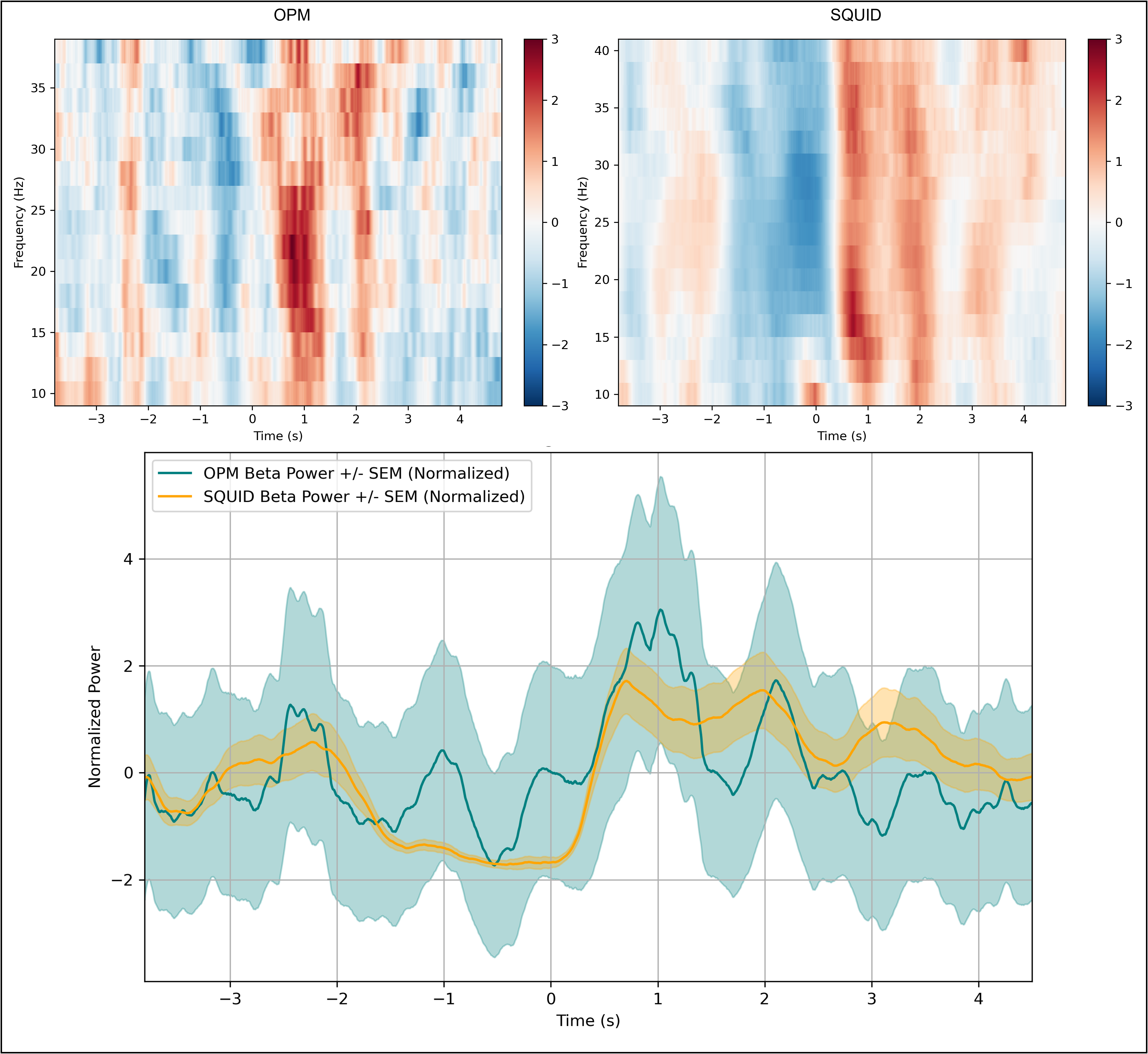


*Figure 5. Time-Frequency Representations (10–40 Hz) and beta power (14–30 Hz) for ALS Patient (Active condition). Top Left: TFR from the OPM session with passive movement, Top Right: TFR from the SQUID session with passive movement. Both TFRs are z-score normalized data. Bottom: Normalized beta power (14–30 Hz) comparison between the two methods (mean ± SEM (shaded area)).*

## *Figure 6: ALS Patient (Passive Condition)*


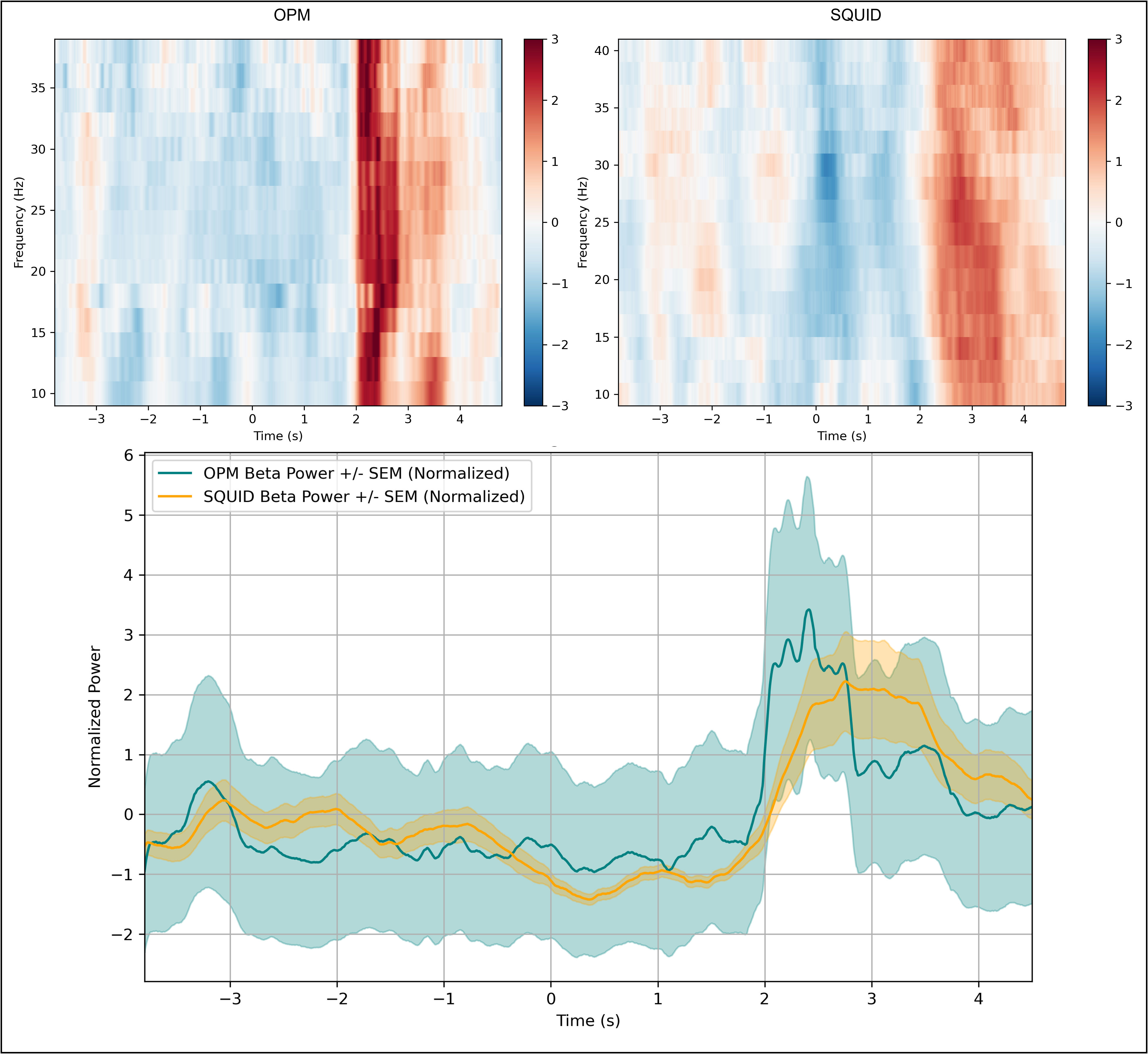


*Figure 6: Time-Frequency Representations (10–40 Hz) and beta power (14–30 Hz) for ALS Patient (Passive condition). Top Left: TFR from the OPM session with passive movement, Top Right: TFR from the SQUID session with passive movement. Both TFRs are z-score normalized data. Bottom: Normalized beta power (14–30 Hz) comparison between the two methods (mean ± SEM (shaded area)).*

## *Figure 7: Healthy Participant 1 (Active Condition)*

*
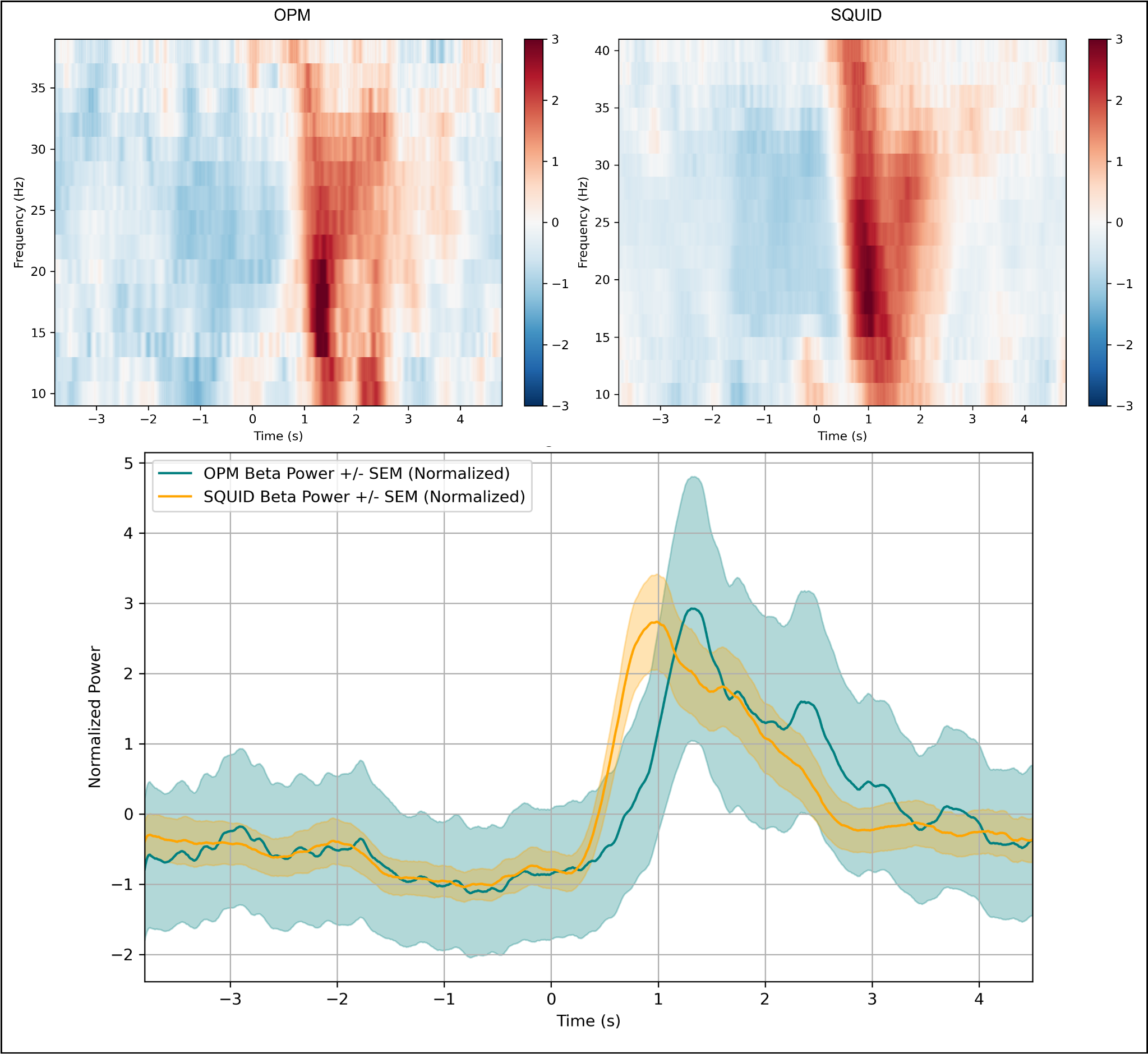
*

*Figure 7: Time-Frequency Representations (10–40 Hz) and beta power (14–30 Hz) for Healthy Participant 1 (Active condition). Top Left: TFR from the OPM session with active movement, Top Right: TFR from the SQUID session with active movement.* *Both TFRs are z-score normalized data. Bottom: Normalized beta power (14–30 Hz) comparison between the two methods (mean ± SEM(shaded area)).*

## *Figure 8: Healthy Participant 1 (Passive Condition)*


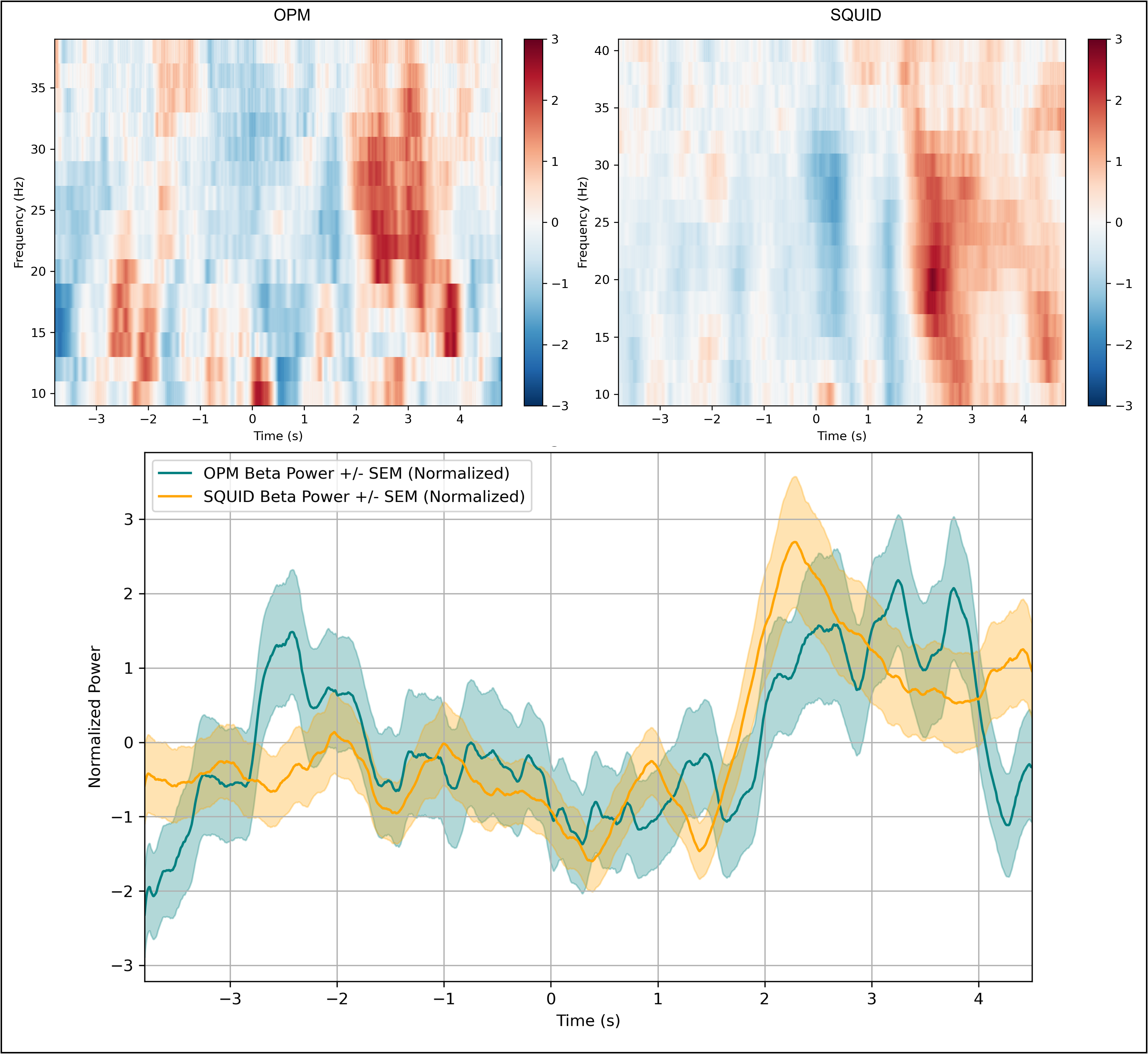


*Figure 8: Time-Frequency Representations (10–40 Hz) and beta power (14–30 Hz) for Healthy Participant 1 (Passive condition). Top Left: TFR from the OPM session with passive movement, Top Right: TFR from the SQUID session with passive movement. Both TFRs are z-score normalized data. Bottom: Normalized beta power (14–30 Hz) comparison between the two methods (mean ± SEM(shaded area)).*

## *Figure 9: Healthy Participant 2 (Active Condition)*

*
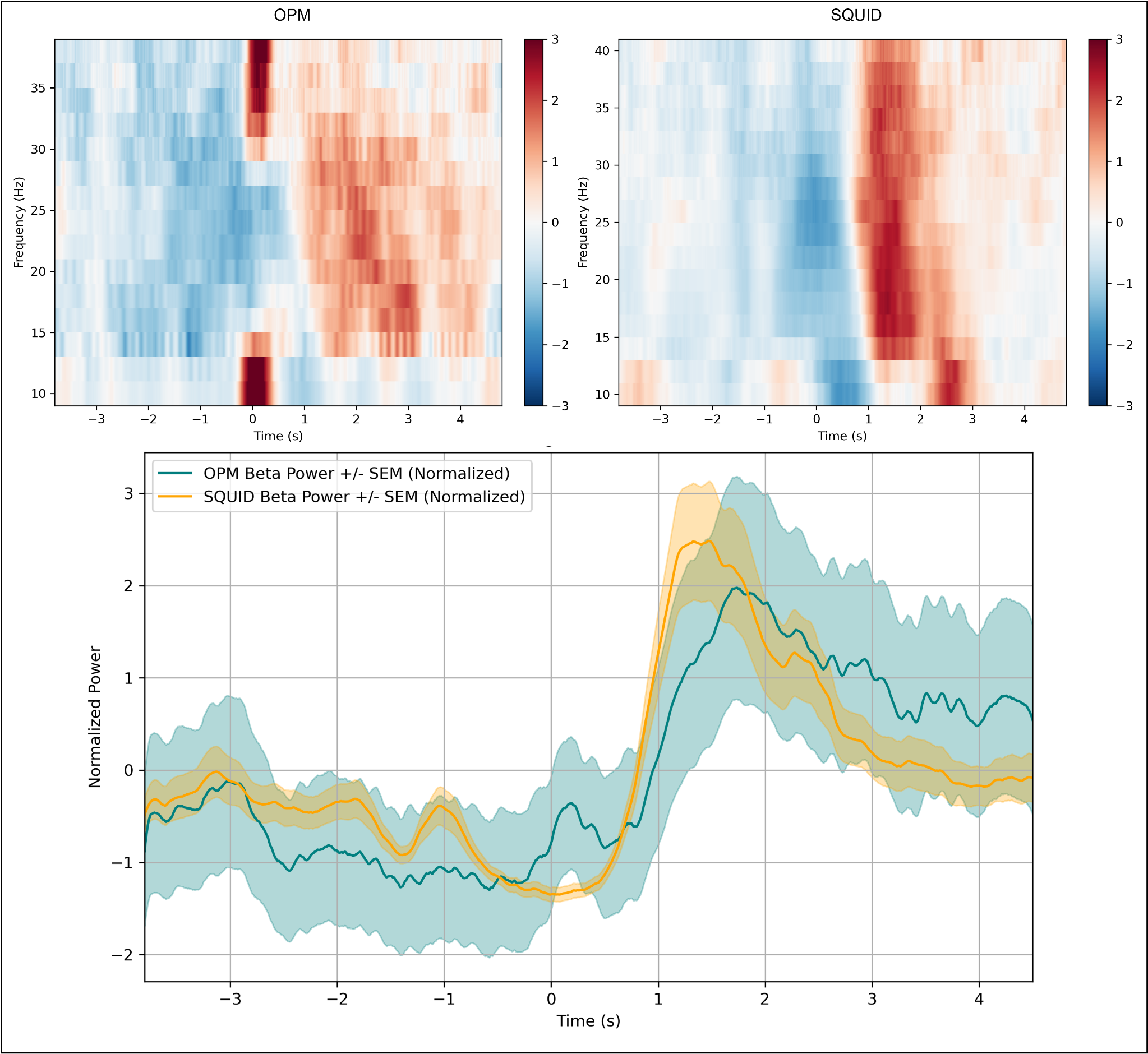
*

*Figure 9: Time-Frequency Representations (10–40 Hz) and beta power (14–30 Hz) for Healthy Participant 2 (Active condition). Top Left: TFR from the OPM session with active movement, Top Right: TFR from the SQUID session with active movement.* *Both TFRs are z-score normalized data. Bottom: Normalized beta power (14–30 Hz) comparison between the two methods (mean ± SEM(shaded area)).*

## *Figure 10: Healthy Participant 2 (Passive Condition)*


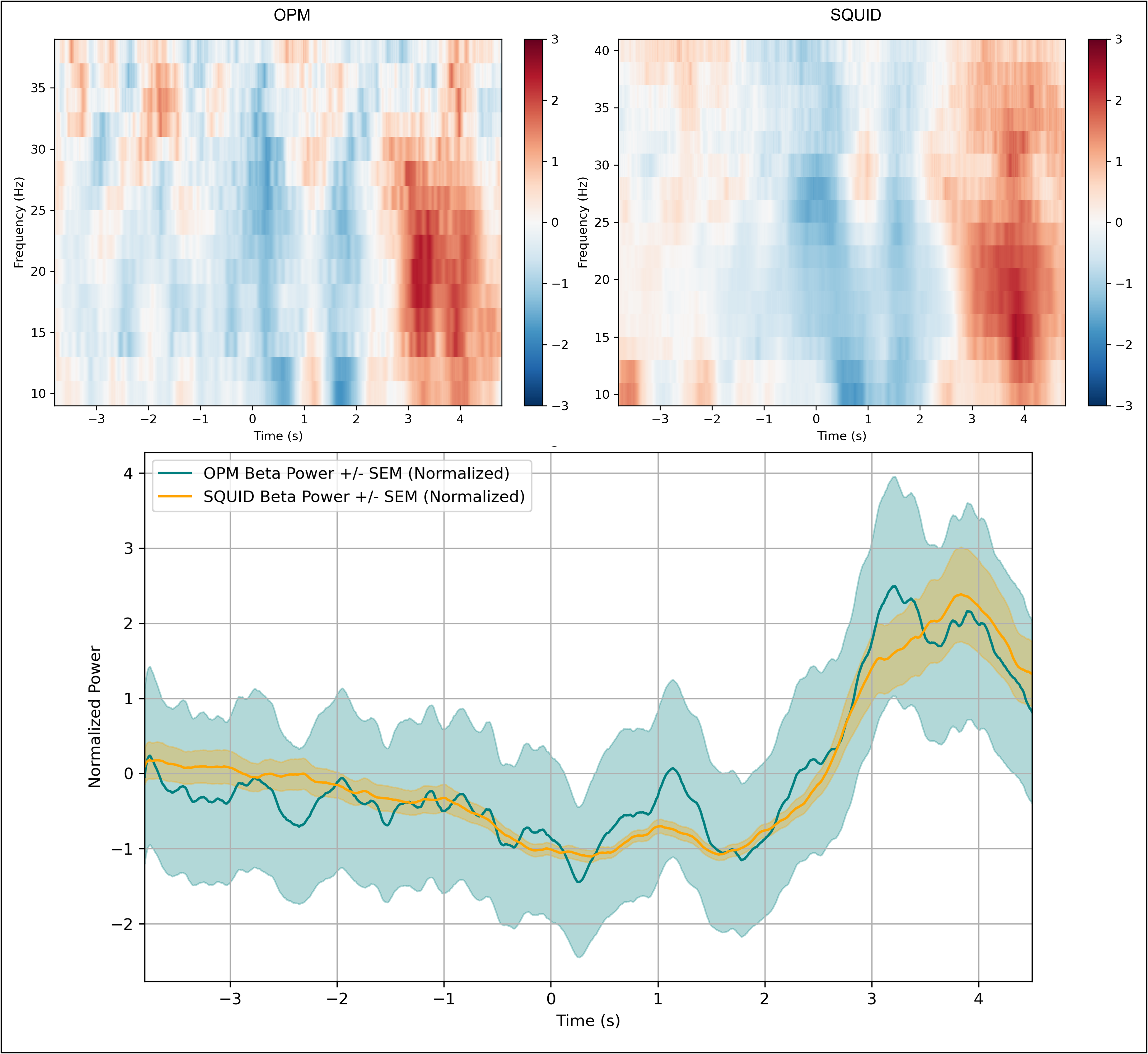


*Figure 10: Time-Frequency Representations (10–40 Hz) and beta power (14–30 Hz) for Healthy Participant 2 (Passive condition). Top Left: TFR from the OPM session with passive movement, Top Right: TFR from the SQUID session with passive movement. Both TFRs are z-score normalized data. Bottom: Normalized beta power (14–30 Hz) comparison between the two methods (mean ± SEM(shaded area)).*

## *Figure 11: Healthy Participant 3 (Active Condition)*

*
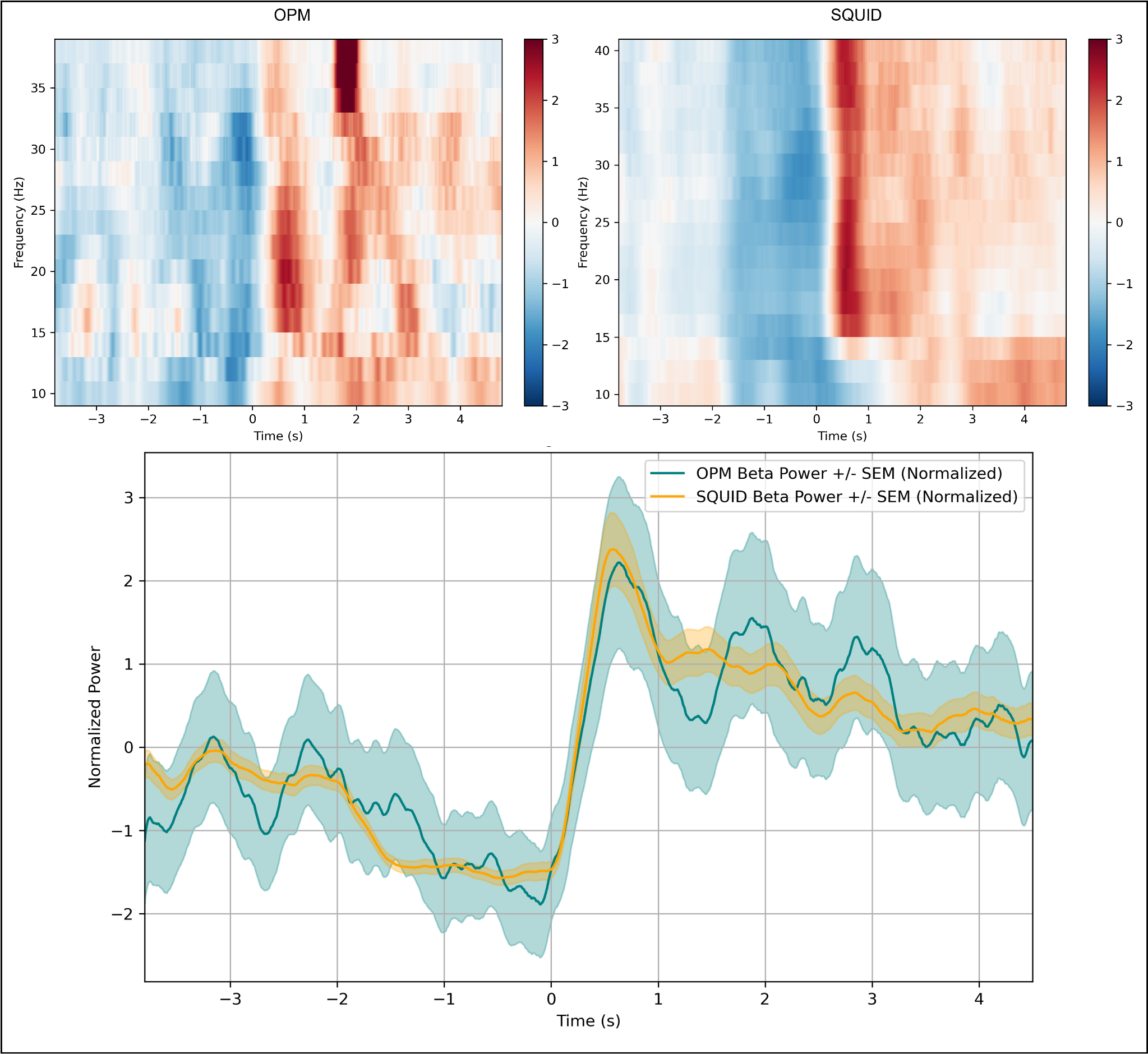
*

*Figure 11: Time-Frequency Representations (10–40 Hz) and beta power (14–30 Hz) for Healthy Participant 3 (Active condition). Top Left: TFR from the OPM session with active movement, Top Right: TFR from the SQUID session with active movement. Both TFRs are z-score normalized data. Bottom: Normalized beta power (14–30 Hz) comparison between the two methods (mean ± SEM(shaded area)).*

## *Figure 12: Healthy Participant 4 (Active Condition)*

*
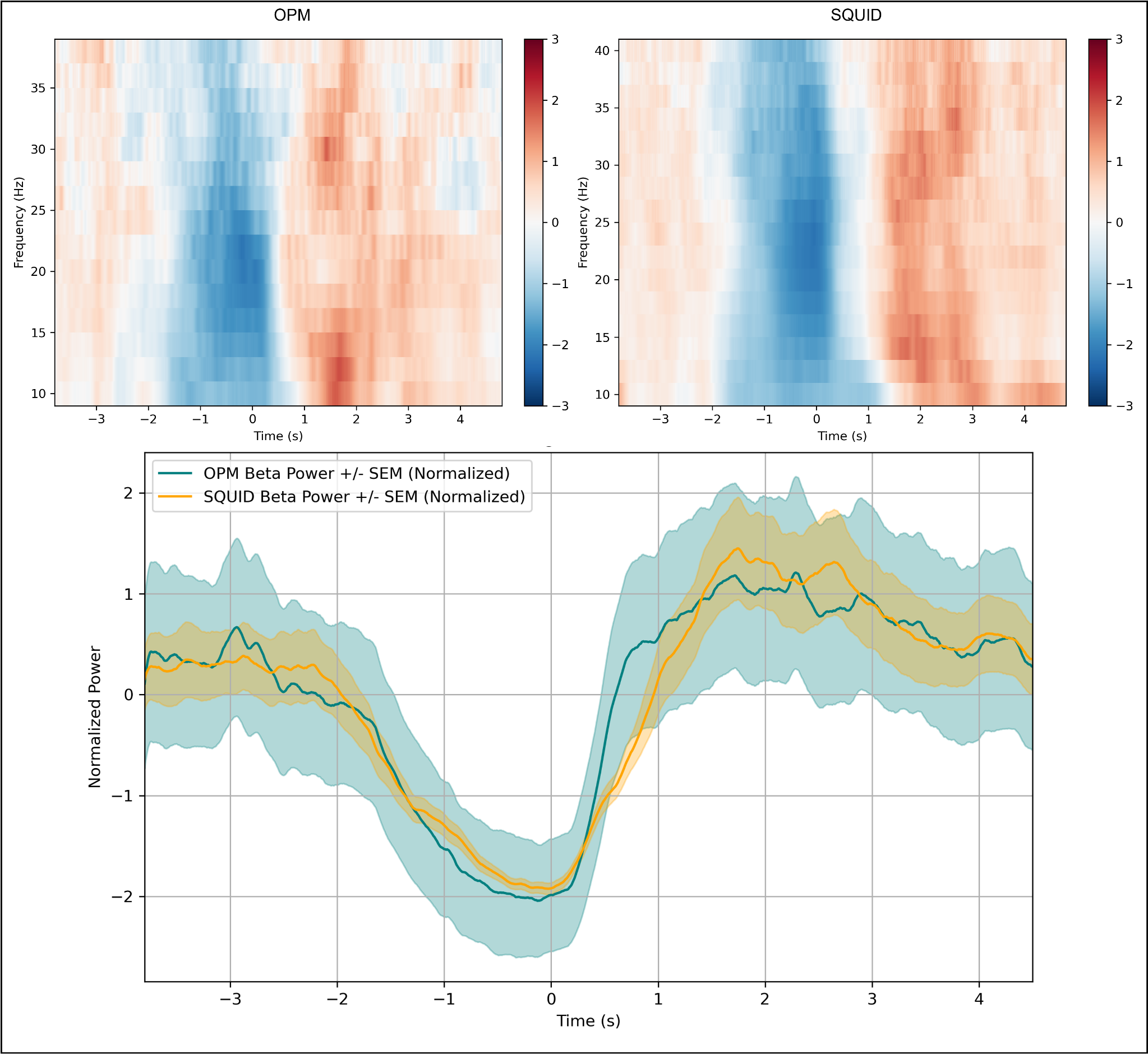
*

*Figure 12: Time-Frequency Representations (10–40 Hz) and beta power (14–30 Hz) for Healthy Participant 4 (Active condition). Top Left: TFR from the OPM session with active movement, Top Right: TFR from the SQUID session with active movement. Both TFRs are z-score normalized data. Bottom: Normalized beta power (14–30 Hz) comparison between the two methods (mean ± SEM(shaded area)).*

## *Figure 13: Healthy Participant 4 (Passive Condition)*


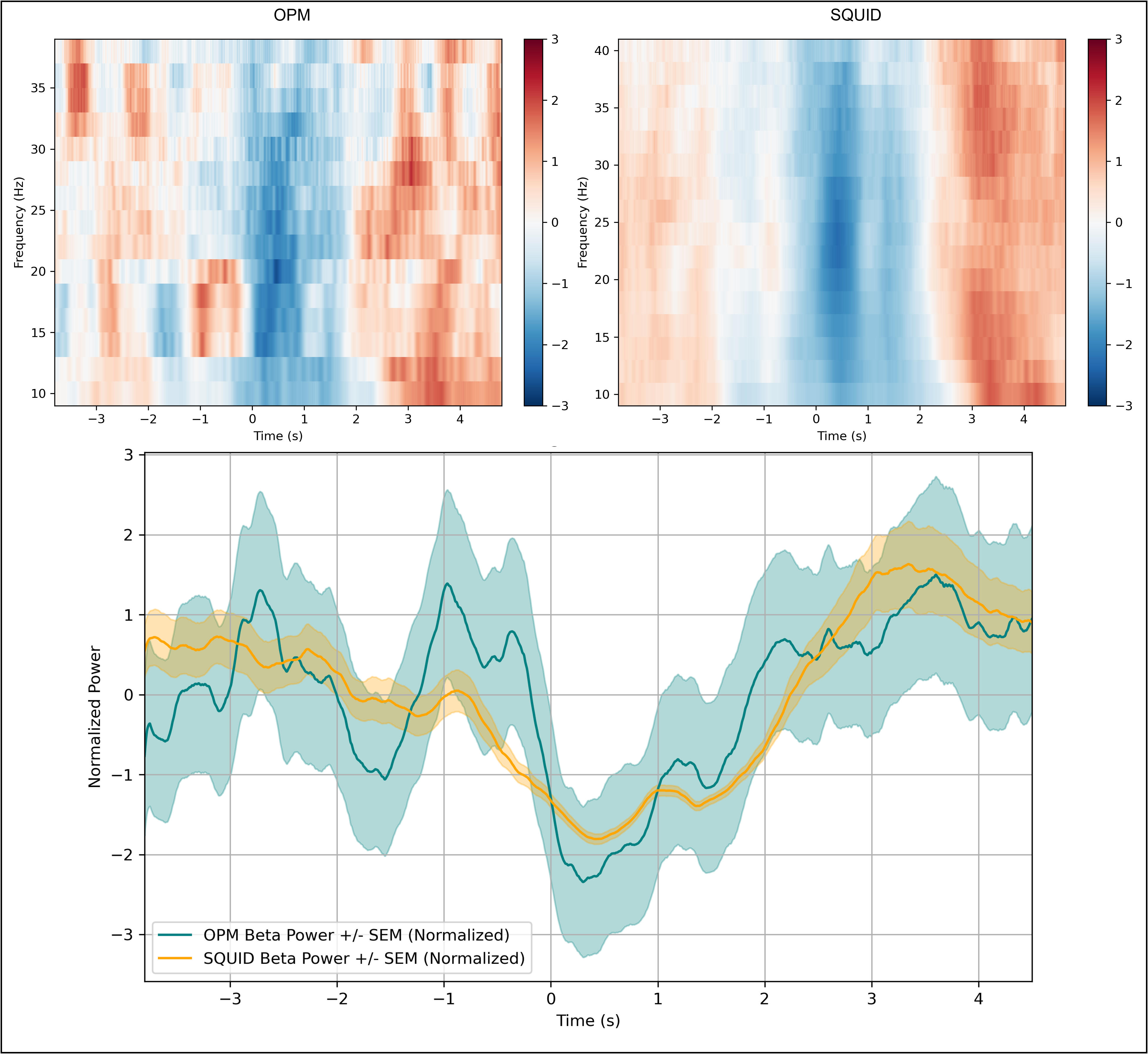


*Figure 13: Time-Frequency Representations (10–40 Hz) and beta power (14–30 Hz) for Healthy Participant 4 (Passive condition). Top Left: TFR from the OPM session with passive movement, Top Right: TFR from the SQUID session with passive movement. Both TFRs are z-score normalized data. Bottom: Normalized beta power (14–30 Hz) comparison between the two methods (mean ± SEM(shaded area)).*
